# Lifetime Corneal Edema Load Model

**DOI:** 10.1167/tvst.10.2.34

**Published:** 2021-02-22

**Authors:** Russell Thomson, Rabia Mobeen, Arthur Ho, Desmond Fonn, Deborah F. Sweeney

**Affiliations:** 1 Western Sydney University, Penrith, NSW, Australia; 2 University of New South Wales, Sydney, NSW, Australia; 3 Brien Holden Vision Institute Limited, Sydney, NSW, Australia; 4 Centre for Ocular Research and Education, University of Waterloo, Waterloo, ON, Canada

**Keywords:** corneal edema, daily disposable contact lenses, silicone hydrogels, hypoxic load

## Abstract

**Purpose:**

To highlight the potential benefits for long-term use of silicone hydrogels daily disposable (DD) contact lenses, particularly with patients who are noncompliant, sleeping or napping while wearing their lenses, or those who have higher oxygen demands and wear this modality for decades.

**Methods:**

Published data for corneal swelling with lenses and no lens wear were used to develop a nonlinear least squares model. The edema load experienced with a range of oxygen transmissibilities (Dk/t) and wear compliance (sleep and napping) was determined. A mixed-effects linear regression model was used to compare the edema load for high and average corneal swellers.

**Results:**

The edema load generated demonstrates that a high Dk/t silicone hydrogel lens results in edema levels close to that with no lens wear. In comparison, hydrogels with a Dk/t of 27 (× 10^−^^9^ [cm mL{O_2_}][s mL mm Hg]), worn on a daily wear schedule will result in 1.5 times more edema and up to two times more if the patient is noncompliant over each decade of wear. High swellers after four decades of wear will have an edema load 10 to 17 times greater than average swellers depending on Dk/t and their degree of noncompliance with the daily wear modality.

**Conclusions:**

Prescribing silicone hydrogel DD lenses, particularly with higher DK/t, may help to maintain the long-term ocular health of patients, when they wear their lenses fulltime for many decades.

**Translational Relevance:**

Illustrates the importance of Dk/t for any CL wear modality where patients nap or sleep in lenses or have high oxygen needs.

## Introduction

The benefits of silicone hydrogels over traditional hydrogel lenses for extended^1^^–^^3^ and daily wear use^3^^–^^5^ to avoid clinical sequelae caused by corneal hypoxia have been well documented_._ Daily disposable (DD) silicone hydrogels have now been available for more than a decade. The uptake of this modality of silicone hydrogel lens wear has increased noticeably in the last few years but not across all markets, and hydrogel DD lens fits have remained steady at about 20% each year.^6^

Numerous studies have established that silicone hydrogel DD lenses provide wearers with comfort, vision, and ease of handling equivalent to hydrogels. DD lenses are no longer predominantly prescribed for part-time wearers, with an international study^7^ reporting that most patients wear their lenses for 14 hours a day seven days a week. Patient noncompliance with DD lens wear can be significant,^8^^–^^10^ with more than a quarter of patients wearing their lenses during sleep and 75% napping regularly during lens wear. It is important that the highest oxygen transmissibility lenses are prescribed for patients who have greater than average oxygen demands^11^^–^^13^ and those who wear higher or more complex prescriptions including torics. Likewise, with the increasing success of presbyopic corrections and the emergence of myopia control contact lenses that are being prescribed to children as young as eight years of age,^14^ patients are likely to be contact lens wearers for many decades, and consideration should be given to prescribing the highest oxygen transmission lens available.

Holden et al.^15^ reported on the corneal consequences of five years of low oxygen transmissible extended wear of hydrogel contact lenses. A limited number of clinical trials involving adults and children have assessed the ocular performance of and rate of adverse events occurring with DD contact lenses over one or so years.^14^^,^^16^^–^^18^ However, to date, no studies have been conducted to evaluate whether there are corneal or other ocular physiological consequences from the long-term daily wear of either hydrogel or silicone hydrogel DD contact lenses.

This study was undertaken to develop a statistical model to determine the long-term edema load associated with DD hydrogel and silicone hydrogel lenses, when worn for several decades and particularly when patients are noncompliant with the modality or have higher oxygen demands. This predicted edema load is compared to that modeled for no lens wear and with extended wear of low oxygen transmissibility hydrogels, to determine whether any long-term corneal consequences might be anticipated after multiple decades or a “lifetime” of DD contact lens wear.

## Methods

### Data

A systematic search of the literature was conducted. The primary source, Medline, Google Scholar, Scopus, PubMed, Embase, and Cochrane Library databases, were searched up to July 2019. No back-date or language restrictions were applied. Search terms “corneal swelling, corneal thickness, corneal edema, overnight, corneal deswelling, hypershoot, diurnal changes, diurnal variation, no lens wear, contact lens wear, open eye, closed eye, eye closure, corneal pachymetry, thinning of the cornea” were used in different combinations. Reference lists of relevant articles were also searched as a secondary source. Published scientific abstracts from the meetings of the Association for Research in Vision and Ophthalmology were also searched.

In total, 318 publications were initially identified to have relevant data. Data from various wear scenarios, including overnight wear, as well as daily wear of soft and silicone hydrogel lens wear, as well as no lens wear, were assembled. Multiple articles from the same author or institution were closely analyzed to ensure the data extracted were not duplicated or accumulated from other studies. Studies involving rigid gas permeable (RGP) or polymethyl methacryte (PMMA) lenses were excluded. Studies were also not included if the subjects had previous lens wear experience^15^ or if there were any pre-existing ocular conditions or previous ocular surgery.^19^ Studies were also excluded if data outcomes were reported in a format that could not be extracted. Studies reporting contralateral lens wear were accounted for based on the work of Fonn et al.^20^ who in neophyte subjects demonstrated that corneal swelling of the contralateral control eyes was significantly lower when there was less swelling of the fellow eye.

On review, lens wear corneal edema data were collated from 31 publications and no lens wear data from 14 publications. Unpublished corneal swelling studies conducted by the authors (Sweeney and Fonn) were included, and where possible data for individual subjects were accessed. This amounted to 561 data points for the modeling of corneal swelling with overnight wear of contact lenses and 512 data points for modeling swelling with no overnight wear. Of these 1073 data points, 64% were based on individual study participants, while the remaining 36% were based on the mean edema of a number of study participants.

Data collated included the number of subjects, age, sex, previous contact lens wear, ocular status, corneal thickness (microns) or corneal swelling (%) at each time (in clock hours) extracted from the text, tables, and graphs (using Engauge Digitizer 10.10), if contact lenses were worn the type of soft or silicone hydrogel contact lens material (s) worn in each eye was noted, as well as back vertex power (in diopters), central and/or average thickness (microns), and center or average oxygen transmissibility (in “barrers” here defined as (× 10^−9^ [cm mL{O_2_}] [s mL mm Hg]) were specified. In addition, the instrument used to monitor corneal thickness was recorded and only data collected by optical or ultrasonic pachymetry were included. Corneal thickness (microns) data were converted to corneal swelling (percentage) for inclusion in the model.

### Model

All analyses were conducted using statistics software: R 3.6.0^21^ (R Core Team).

#### Clinical Assumptions

The following assumptions, based on clinical insight and/or the literature, were included in the model:

1. Lenses were worn for 14 hours a day, seven days a week^7^.
2. Lenses were inserted one hour after waking from eight hours of sleep.
3. Lenses were worn while patients slept^7^^,^^9^; lenses remained in situ during sleep and were removed 15 hours after waking on Day 2.
4. Lenses were worn when patients napped^7^^,^^9^; napping occurred in the early afternoon for one hour with lenses in situ, lenses were removed one hour before sleep on the same day.
5. Overnight swelling response followed a normal distribution for both non-lens and lens wear.^13^
6. The swelling response of individuals is independent of lens type and can be categorized as low, medium, or high.^22^
7. The swelling response is consistent over time.

#### Rate of Overnight Corneal Swelling

The data available included overnight swelling data for several lens types and with no lens wear. There were no data available on the rate of corneal swelling overnight with or without lens wear.

Data describing the swelling curves for different levels of atmospheric oxygen over an eight-hour period with different mixtures of gas introduced to the eye by goggles^23^ for eight individuals were available. A nonlinear least squares (NLS) model of these data was used to estimate the overnight swelling curves for both lens wear and with no lens wear.

(1) $$Overnight\;Swelling\;=\;\frac{A.log\left(B_{h}t+1\right)}{oxygen}$$

The measured variable, *t*, represents the number of hours after waking, and *oxygen* represents the level of oxygen available to the closed eye. The coefficients, *A* (representing the total swelling observed relative to oxygen availability) and *B_h_* (representing the rate of swelling over time), were estimated from the data (Table 1).

**Table 1.** Parameter Estimates From Equation 1, for the Total Corneal Swelling

Variable	Estimate	SE	t Value	Pr(>|t|)
**A**	1.27	0.55	2.31	0.028
**B_h_**	13.6	23.7	0.576	0.57

The parameter, *A* represents the total swelling observed relative to oxygen and the parameter, *B_h_* represents the rate of swelling over time.

The overnight swelling curves, as a result of overnight wear of a contact lens of given Dk/t, was estimated by equating Equation 1 with Equation 2 when t = 0, and solving for *oxygen*. This level of *oxygen* was then used in conjunction with Equation 1 to estimate the rate of corneal swelling overnight for a lens of a given Dk/t.

#### Corneal Deswelling After Sleep With and Without Lens Wear

Only limited data were available on the rate of deswelling following overnight lens wear when the lens is worn for the following day for 14 hours. Holden, Mertz and McNally^24^ monitored the corneal thickness variation across seven days of continuous wear of three different hydrogel lenses. Data were reported for days 2 through 7 on eye-opening after eight hours of sleep and at one, five, and 12 hours after eye-opening. The mean corneal deswelling for the group of subjects over the 12 hours after sleep was both limited and reasonably consistent for all lens types (8.2% ± 1.1%). Consequently, the level of daytime corneal edema was determined primarily by the overnight swelling and the amount of deswelling was on average 8% over the 12-hour period of lens wear after eye-opening.

An NLS model was used to model deswelling from the point of waking and throughout the remainder of the day (Equations 2–4). Equation 2 gives the deswelling rate after overnight sleep while wearing lenses. The measured variable, *t* is the number of hours after eye-opening. For some studies, the study participants had different lenses in each eye. Measured variables, *D_k/t_*and *cD_k/t_*are the oxygen transmissibility of the lens in the ipsilateral and contralateral eye, respectively.

(2) $$\begin{array}{c} \begin{array}{c} Deswelling_{after\;sleep}=\\ \left(B_{nap}+B_{diff}\right)e^{-(D_{k/t}*\left(k_{D}+k_{2}*t)+cD_{k/t}*\left(kc_{D}+kc_{2}*t\right)+k_{deswell}*t\right)}\;\\ +\left(L_{0}+L_{diff}\right)\left(\frac{1}{{\left(D_{k/t}\right)}^{b}}-\frac{1}{{\left(D_{N}\right)}^{b}}\right) \end{array} \end{array}$$

The coefficient, *D_N_* indicates the D_k/t_ of a lens that has the same swelling pattern of a study participant with no lens wear. *D_N_* was assumed to be higher than the highest Dk/t of a lens observed in the data set.

(3) $$\begin{array}{ccc} & & Daytime\;Swelling_{After\;lens\;insertion}\\ & & =\left(L_{0}\right)\left(\frac{1}{{\left(D_{k/t}\right)}^{b}}-\frac{1}{{\left(D_{N}\right)}^{b}}\right)\log\left(k_{day}*t+1\right) \end{array}$$

#### The Effect of Napping

Hamano et al.^25^ monitored corneal swelling over an 8-hour period during the day with a 1-hour nap in the early afternoon. These data were used to model the level of swelling after a 1-hour nap, and the deswelling afterward (Equation 4).

(4) $$\begin{array}{c} \begin{array}{c} Deswelling_{After\;Nap}\\ =\left(B_{nap}\right)e^{-(D_{k/t}*\left(k_{D}+k_{2}*t)+cD_{k/t}*\left(kc_{D}+kc_{2}*t\right)+k_{deswell}*t\right)}\\ +\left(L_{0}+L_{diff}\right)\left(\frac{1}{{\left(D_{k/t}\right)}^{b}}-\frac{1}{{\left(D_{N}\right)}^{b}}\right) \end{array} \end{array}$$

Equations 2, 3 and 4 were fitted using a single NLS model. Data from deswelling after overnight wear, deswelling after a nap, deswelling with no lenses, and swelling after lens insertion in the morning, were all used to estimate the parameters across these equations. The equations were combined with the use of indicator functions. The term $\left(L_{0}+L_{diff}\right)\left(\frac{1}{{\left(D_{k/t}\right)}^{b}}-\frac{1}{{\left(D_{N}\right)}^{b}}\right)$ represents the residual swelling at the end of the day. These equations were developed through consultation between clinical experts and the modelers. For example, this term was included to represent what has been observed clinically.

#### Calculating the Edema Load

Corneal swelling was estimated over a 24-hour period for a range of lenses, using the NLS models, based on the following scenarios:

1. No lens wear with eight hours sleep.
2. Daily wear (compliant) where the patient sleeps for 8 hours, insert their lenses one hour after waking, wears the lens for 14 hours before going to sleep.
3. Daily wear (noncompliant), where the patient sleeps for eight hours, inserts their lenses one hour after waking, wears their lenses for 15 hours including a one-hour nap in the early afternoon.
4. Extended wear (noncompliant) where the patient sleeps for eight hours, inserts their lenses one hour after waking and does not remove their lenses until the following day after eight hours of sleep and 15 hours of open eyewear.

Estimates were made for lenses with oxygen transmissibilities of 11, 27, 100, and 157.

The area under the swelling curves was used to determine the edema load over a 24-hour period for no lens wear and compliant and noncompliant lens wear for each of the selected oxygen transmissibilities. These estimates were then extended to calculate the edema load over 10, 20, 30, or 40 years.

#### Model of High Swellers

Moezzi et al.^22^ showed significant between-subject variation, concluding that there are low, medium and high swellers. For this article, high swellers are defined as the highest sweller of 10 random individuals wearing the same lenses with the same level of compliance. The modeling of between-subject variation in swelling was undertaken using a linear regression model, with normally distributed errors and subjects treated as a random effect, using the *R* package lme4. The dependent variable used was the residuals from the NLS equations. The data for this model was limited to studies where individual data were given.

## Results

**Figure 1.**
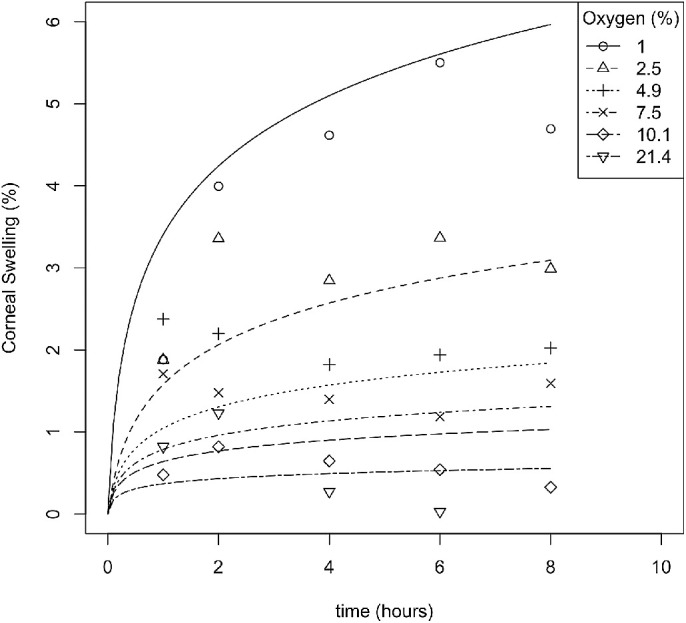
Predicted curves of swelling overnight, for different levels of oxygen available to the eye. Data obtained from a study that used different mixtures of gas introduced to the eye by goggles.^23^ Predictions are based on an NLS model with Equation 1 and parameters given in Table 1.

**Figure 2.**
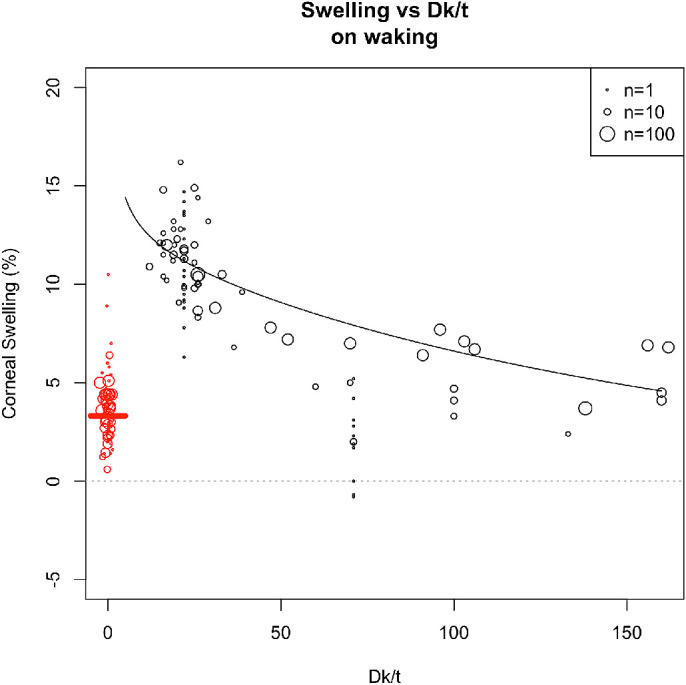
Relationship between *Dk/tc* and corneal swelling at eye opening (after wearing the lens overnight). The *black line* is derived from the NLS model, using the estimated parameters in Table 2. The *circles* show the data points from various studies, with the sample size of the study indicated by the size of the *circle*. The *red circles* indicate the data when no lens has been worn. The *red line* shows the estimate of overnight swelling with no lens wear. The black data points (*circles*) are positioned according to the *Dk/tc* in the ipsilateral eye (ignoring the Dk/t of the lens in the contralateral lens), whereas the *black line* is based on estimated swelling as a result of wearing lenses with the same *Dk/tc* in both eyes.

The NLS coefficients for Equation 1 are presented in Table 1 and the model fits are presented in Figure 1. The coefficients for Equations 2, 3 and 4, are presented in Table 2 and the model fits are presented in Figure 2. A contralateral effect on the corneal swelling of the fellow eye has been previously reported.^20^ The model estimates the overnight swelling of a person wearing a lens (of D_k/t_ = 11) in the ipsilateral eye only, the contralateral eye only or in neither eye to be 10.8%, 3.9%, or 3.3%, respectively.

**Table 2.** Estimated Coefficients From the NLS Model, Based on Equations 2, 3 and 4

Variable	Estimate	SE	t Value	Pr(>|t|)
B_nap_	5.12	0.795	6.44	<0.0001
B_diff_	6.32	0.796	7.95	<0.0001
k_deswell_	0.653	0.0631	10.3	<0.0001
k_D_	0.00608	0.000579	10.5	<0.0001
kc_D_	0.000977	0.000281	3.47	0.00054
k_2_	0.00127	0.0017	0.745	0.46
kc_2_	0.0034	0.00139	2.44	0.015
D_N_	177	13.4	13.2	<0.0001
L_0_	1.37	0.514	2.67	0.0077
L_diff_	8.62	0.722	11.9	<0.0001
k_day_	83.2	181	0.46	0.65

*B_nap_* and *(B_nap_+B_diff_)* indicate the level of swelling at eye opening after a nap and sleep respectively. *k_deswell_* indicates the rate of de-swelling over time. *k_D_* and *kc_D_* represent the relationship between de-swelling and D_k/t_. *k_2_* and *kc_2_* represent the relationship between de-swelling and the interaction between D_k/t_ and time. The *c* in these coefficients are for de-swelling in the contralateral eye. *D_N_* indicates the equivalent D_k/t_ for the de-swelling pattern of no lens wear. *L_0_* and *(L_0_+L_diff_)* indicate the level of residual swelling at the end of the day, following overnight wear and no overnight wear respectively.

### Modeling 24-Hour, Two-Week, and “Lifetime” Swelling Patterns

**Figure 3.**
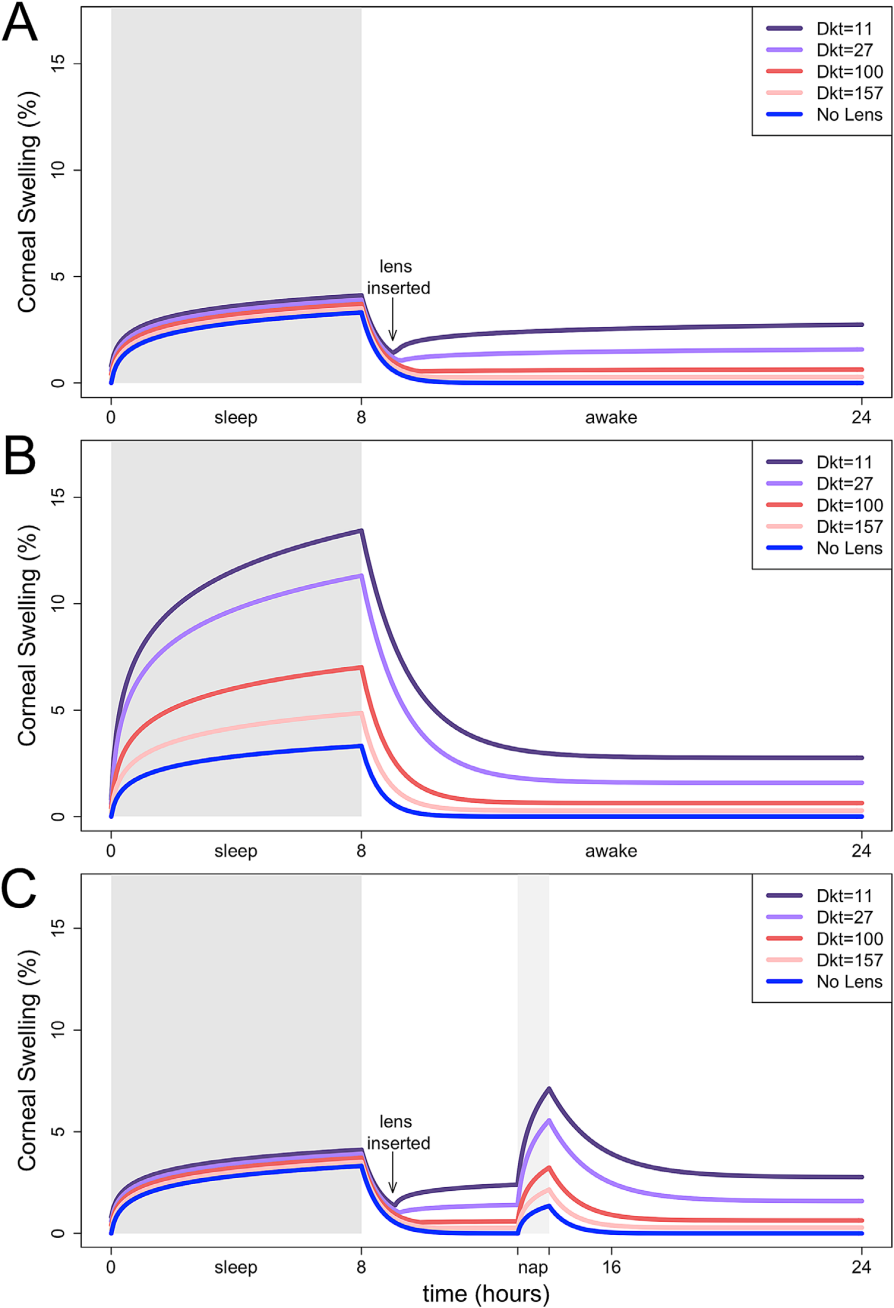
The estimated 24-hour pattern of swelling with different lens wear scenarios. (A) Daily wear lens inserted one hour after waking. (B) Extended wear lens worn overnight and the next day. (C) Daily wear; lens inserted one hour after waking and continued to wear during a one-hour nap. Sleep and nap areas indicated by *dark* and *light gray shade*, respectively.

**Figure 4.**
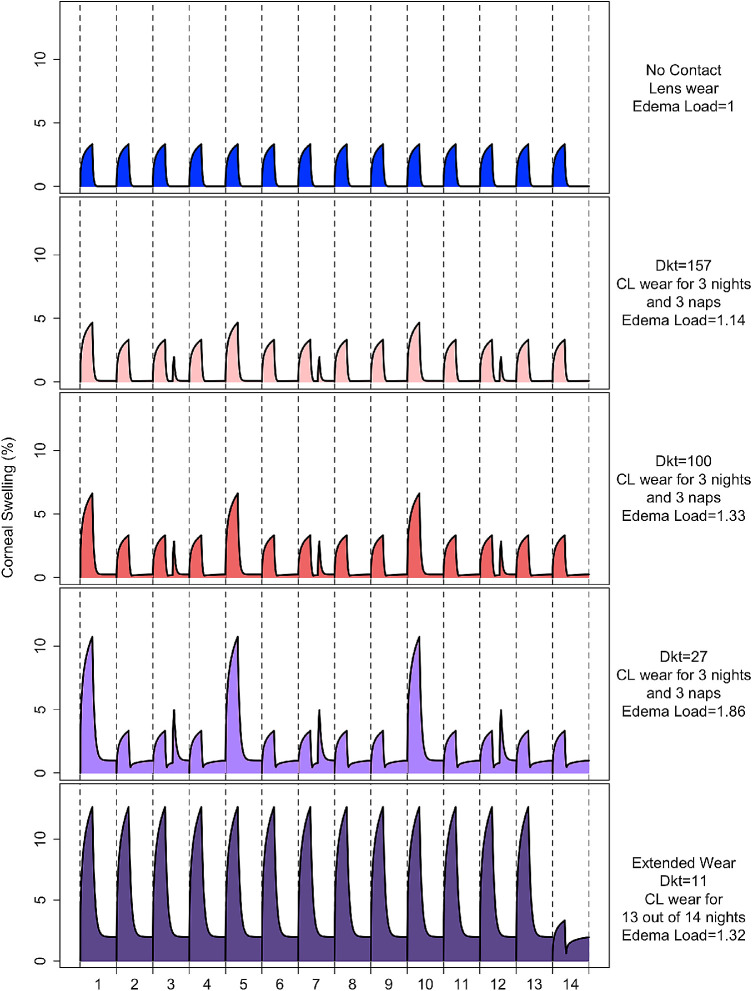
The edema load under different scenarios, as indicated by the area under the curve, over a two-week period. The edema load units are relative to the edema load with no contact lens wear for two weeks.

The edema load was calculated for the various wear scenarios and lens options, including no lens wear (Fig. 3). ^1^,^2^,^3^Figure 4 depicts a range of scenarios as an example over a two-week period.

Table 3 presents the edema load for a typical patient under different wear scenarios with different Dk/t lenses for 10 to 40 years of lens wear, relative to the edema load of a typical patient who does not wear a lens for 10 years. The edema load calculated for a high Dk/t (157) lens is similar to “load” with no lens wear. In comparison, a lens with Dk/t of 27 will result in 1.5 times the edema load compared to no lens wear over each decade of wear.

**Table 3.** The Mean Edema Load for a Typical Patient Under Different Wear Scenarios With Different Dk/Tc Lenses for 10 to 40 Years of Lens Wear, Relative to the Edema Load of a Typical Patient Who Does Not Wear a Lens for 10 Years

					One Nap and One				Eight Naps and Four			
Lens Type/DK/t	No Sleep or Nap				Sleep in Lens/Month				Sleeps in Lens/Month			
Years of Wear	10	20	30	40	10	20	30	40	10	20	30	40
Low Dk hydrogel 10	2	4.1	6.1	8.2	2.2	4.3	6.5	8.7	2.6	5.1	7.7	10.3
27	1.5	2.9	4.4	5.8	1.5	3.1	4.6	6.2	1.8	3.7	5.5	7.4
100	1.1	2.2	3.2	4.3	1.1	2.2	3.3	4.5	1.2	2.4	3.6	4.9
157	1	2	3	4.1	1	2	3.1	4.1	1	2.1	3.1	4.2

There is little difference in the edema load that a patient fitted with a high Dk/t lens experiences regardless of their compliance with the daily wear modality (i.e., does not wear their lenses while they sleep or nap versus those who wear their lenses while sleeping four nights, as well as napping while wearing lenses on 8 occasions per month) and this difference does not increase significantly with many decades of wear. In comparison, if a patient wears a conventional hydrogel (Dk/t = 27), there is a substantial increase in edema load over multiple decades of noncompliance compared to the edema load with a high Dk/t (157) silicone hydrogel.

The effects of the edema load experienced by both compliant and non-compliant wearers of hydrogel and silicone hydrogel DD lenses can be estimated by comparing the edema load to that experienced with a low Dk (10) hydrogel worn on a continuous wear basis (13 out of 14 nights every two weeks).

In Table 4, a compliant patient wearing a DD hydrogel or silicone hydrogel for 10 years has an edema load of 0.6 or 0.4 times, respectively, of that associated with five years of continuous extended wear of a low Dk/t hydrogel. In comparison, after 40 years of noncompliant wear of a hydrogel disposable lens, on average the edema load is three times that of a wearer of a low Dk/t hydrogel continuously (13 out of 14 nights) for five years. With a high Dk/t silicone hydrogel with noncompliant wear, the edema load is 1.7 times greater and similar to that seen with no lens wear over the 40-year period.

**Table 4.** The Mean Edema Load for a Typical Patient Under Different Wear Scenarios With Hydrogels and Silicone Hydrogels of Different Dk/T Lenses for 10 to 40 Years of Lens Wear, Relative to the Edema Load of a Typical Patient Who Wears Low Dk/T Hydrogels Continuously (13 of 14 Nights) for 5 Years

					One Nap and One				Eight Naps and Four			
Lens (DK/t)	No Sleep or Nap				Sleep in Lens/Month				Sleeps in Lens/Month			
Years of Wear	10	20	30	40	10	20	30	40	10	20	30	40
Conventional hydrogel (Dk/t 27)	0.6	1.1	1.7	2.3	0.6	1.2	1.8	2.4	0.7	1.5	2.2	3
Silicone hydrogel (Dk/t 100)	0.4	0.9	1.3	1.7	0.4	0.9	1.3	1.8	0.5	1	1.5	2
Silicone hydrogel (Dk/t 157)	0.4	0.8	1.2	1.6	0.4	0.8	1.2	1.6	0.4	0.8	1.3	1.7
No lens	0.4	0.8	1.2	1.6	0.4	0.8	1.2	1.6	0.4	0.8	1.2	1.6

Data for no lens wear are also included as a reference.

Table 5 presents the edema load for the high sweller compared to the edema load for an average sweller. This illustrates that the edema load of the high sweller, for both compliant and noncompliant wear, increases as the Dk/t of the lens reduces.

**Table 5.** The Edema Load for the Highest Sweller of 10 Patients Under Different Wear Scenarios With Different Dk/Tc Lenses for 10 to 40 Years of Lens Wear, Relative to the Edema Load of an Average Patient Who Does Not Wear a Lens for 10 Years

					One Nap and One				Eight Naps and Four			
Lens (DK/t)	No Sleep or Nap				Sleep in Lens/Month				Sleeps in Lens/Month			
Years of Wear	10	20	30	40	10	20	30	40	10	20	30	40
Low Dk hydrogel (10)	3.6	7.3	10.9	14.5	3.8	7.5	11.3	15.1	4.2	8.5	12.7	16.9
Conventional hydrogel (27)	3.1	6.1	9.2	12.2	3.1	6.3	9.4	12.6	3.5	7	10.5	14
Silicone hydrogel (100)	2.7	5.4	8	10.7	2.7	5.4	8.1	10.9	2.8	5.7	8.5	11.4
Silicone hydrogel (157)	2.6	5.2	7.8	10.4	2.6	5.2	7.9	10.5	2.7	5.3	8	10.7
No lens	2.6	5.2	7.8	10.4	2.6	5.2	7.8	10.4	2.6	5.2	7.9	10.5

## Discussion

Silicone hydrogels contact lenses with high oxygen transmission are now available for extended, reusable daily and DD wear. DDs are widely prescribed for full-time wearers with up to 14 hours of wear a day, seven days a week.^7^ With a striking increase in the numbers of presbyopes^6^ wearing lenses plus myopia control gaining increasing acceptance,^6^ we should expect that our patients could commence lens wear in their teens and remain full-time contact lens wearers for 20, 30, or even 40-plus years or wear lenses for presbyopia for two to three decades. Open eye oxygen requirements of patients wearing their contact lenses for most waking hours every day of the week for multiple decades is still of concern. Noncompliance with wear schedule instructions, namely napping or sleeping in daily wear or DD lenses, necessitates the consideration of the higher closed eye oxygen needs be considered.

An international survey of practitioner's perceptions found they believe that DD silicone hydrogel wearers have lower dropout rates than wearers of other types of contact lenses, and 92% agree that silicone hydrogel DDs are the best choice to safeguard eye health related to contact lens wear.^26^ However, there have been no long-term evaluations of the potential corneal physiological consequences of either hydrogels or silicone hydrogels when used as DDs. This edema load model was developed to be able to predict and compare the amount of edema with lenses of varying oxygen transmissibilities that would be produced with decades of wear particularly when patients are non-compliant with the modality.

The edema load results generated demonstrate that a silicone hydrogel lens with a high oxygen transmissibility (Dk/t ≥100), similar to that required to avoid corneal edema with closed eye lens wear, worn on a daily wear mode, results in edema levels close to that with no lens wear. In comparison, hydrogel lenses with a Dk/t of 27, worn on a daily wear schedule, will result in 1.5 times more edema.

The noncompliant habits of DD wearers include importantly from an edema model perspective, patients frequently wearing their lenses during sleep or napping. Patients under 25 years of age are twice as likely to wear their lenses during sleep than older patients,^7^ with almost 2% of patients reportedly wearing their DDs every night during sleep.^9^ Up to 75% of patients^7^^,^^9^ reported occasional napping in their DD lenses with the median number of days for napping while wearing DD contact lenses in the preceding month being two.^7^ It has also been demonstrated that as the number of years of lens wear increase, non-compliance increases.^18^ Noncompliance is defined here as regular sleeping or napping with lenses. Further extensions to this research would be to predict edema load for varying scenarios of non compliance.

The model allows comparisons to occur when a patient does not follow the recommended wear instructions and is noncompliant by either napping or sleeping in their lenses infrequently or more regularly. For example, although little difference is indicated with a high (≥100) Dk/t lens for when a patient may sleep one night every week and have a short nap two days a week in their lenses compared to no lens wear, the edema load is nearly double for a low Dk/t (27) lens.

The edema load comparisons for daily wear were made assuming the participants wear their lenses for 14 hours a day. Further extensions to this study could be made through predicting a range of wear patterns, by using the parameter estimates in Table 2, and applying them to Equation 3.

It is well established that extended wear of conventional hydrogels presents a considerable physiological challenge to the human cornea.^15^ To indicate the corneal consequences of the long-term edema load associated with DDs, the load was compared to the calculated edema load for five years of a low Dk high water content hydrogel (Dk/tc 10/11) extended wear that has been documented to result in significant changes to all layers of the cornea. The conventional hydrogels, even when worn compliantly on a daily wear basis could result in significant changes in at-risk patients to their corneal function over decades of wear. This model suggests that compliant wear of a conventional hydrogel on a DD basis for 40 years produces an edema load equivalent to approximately 12 years of continuous wear of a low Dk/t high water content hydrogel.

As expected, the edema load increases when the patient is noncompliant with the wear schedule. Although noncompliance with the DD wear schedule is not recommended, regular sleeping or napping while wearing high Dk/t silicone hydrogels causes little increase in the predicted edema load after 40 years and is equivalent to approximately seven to eight years of low Dk/t hydrogel extended wear. It would therefore be anticipated that the long-term use of high Dk/t silicone hydrogels DD lenses will cause less physiological changes to the cornea than conventional hydrogel DDs. These benefits will be greater for those patients that do not adhere to the recommended wear schedule or inadvertently sleep or nap in their lenses.

The corneal swelling responses of individuals to overnight sleep, as well as contact lens wear, have been demonstrated to vary independently of the lens^22^ and can be categorized as low, medium, or high swellers. The edema load estimated for the highest sweller of ten individuals demonstrates that high Dk/t lenses moderate edema levels and the predicted hypoxic consequences, especially when lens wear is for decades and involves noncompliant lens wear. Given the results of this model and the increased use of myopia control lenses for younger patients, the introduction of silicone hydrogel lenses for myopia control in the coming years will be of great benefit.

The statistical model in this article was developed via expert opinion and applied to data from past experiments. It is possible the results could be biased by small, poorly designed experiments. To minimize bias, raw data were collected from the authors where possible. Where this was not always possible, studies were weighted by the inverse of the sample size.

The validation of this statistical model of the long-term edema load would require either a prospective clinical trial or a retrospective analysis as was conducted by Holden et al.^15^ to evaluate whether there are any corneal or other ocular physiological consequences from the long-term daily wear of either hydrogel or silicone hydrogels DD contact lenses.

## Conclusion

The differences predicted in the edema load for a typical as well as high swelling patients between conventional and silicone hydrogel DD lenses are marked. Compared to hydrogel DDs, prescribing silicone hydrogel DD lenses, particularly those with higher oxygen transmissibility, will help maintain the long-term ocular health of patients, when lenses are worn for up to 14 or so hours a day, seven days a week for many decades. This advantage is of importance when patients sleep and nap in their lenses on a regular basis. Silicone hydrogel DDs should be considered as the contact lens of choice when fitting younger patients, especially for patients with high prescriptions or those requiring toric designs, given that they have the potential to be lens wearers for many decades.
